# The ATP Synthase γ Subunit ATPC1 Regulates RNA Editing in Chloroplasts

**DOI:** 10.3390/ijms24119203

**Published:** 2023-05-24

**Authors:** Jia Ni, Wenjian Song, Nadia Ahmed Ali, Yayi Zhang, Jiani Xing, Kexing Su, Xingxing Sun, Xiaobo Zhao

**Affiliations:** Key Laboratory of Nuclear Agricultural Sciences of Ministry of Agriculture and Rural Affairs, Key Laboratory of Nuclear Agricultural Sciences of Zhejiang Province, Institute of Nuclear Agricultural Sciences, College of Agriculture and Biotechnology, Zhejiang University, Hangzhou 310058, China; nijia@zju.edu.cn (J.N.); viencentsong@zju.edu.cn (W.S.); nadiaahmed@zju.edu.cn (N.A.A.); yayizhang@zju.edu.cn (Y.Z.); xingjiani@zju.edu.cn (J.X.); dora.skx@zju.edu.cn (K.S.); sunxingxing@zju.edu.cn (X.S.)

**Keywords:** RNA editing, atpC1, MORFs, chloroplast, protein interaction

## Abstract

RNA editing is the process of modifying RNA molecules by inserting, deleting, or substituting nucleotides. In flowering plants, RNA editing occurs predominantly in RNAs encoded by the organellar genomes of mitochondria and chloroplasts, and the main type of editing involves the substitution of cytidine with uridine at specific sites. Abnormal RNA editing in plants can affect gene expression, organelle function, plant growth, and reproduction. In this study, we report that ATPC1, the gamma subunit of ATP synthase in *Arabidopsis* chloroplasts, has an unexpected role in the regulation of editing at multiple sites of plastid RNAs. The loss of function of *ATPC1* severely arrests chloroplast development, causing a pale-green phenotype and early seedling lethality. Disruption of *ATPC1* increases the editing of *matK*-640, *rps12*-i-58, *atpH*-3′UTR-13210, and *ycf2*-as-91535 sites while decreasing the editing of *rpl23*-89, *rpoA*-200, *rpoC1*-488, and *ndhD*-2 sites. We further show that ATPC1 participates in RNA editing by interacting with known multiple-site chloroplast RNA editing factors, including MORFs, ORRM1, and OZ1. The transcriptome in the *atpc1* mutant is profoundly affected, with a pattern of defective expression of chloroplast development-related genes. These results reveal that the ATP synthase γ subunit ATPC1 is involved in multiple-site RNA editing in *Arabidopsis* chloroplasts.

## 1. Introduction

RNA editing is a phenomenon in which transcriptionally processed RNA molecules are modified by nucleotide insertions, deletions, or replacements, leading to changes in the genetic information contained within RNA [1,2]. RNA editing is an important post-transcriptional gene expression regulatory mechanism and RNA modification process, that is designed to correct deleterious mutations inherited from genomes rather than produce protein polymorphisms [3,4], and it is found in many species [5,6]. RNA editing in flowering plants is unique; it predominantly occurs in RNAs encoded by the important energy metabolism organelles—chloroplasts and mitochondria—with the main type of editing involving the conversion of cytidines (C) to uridines (U) [4,7]. Generally, RNA editing can convert about 20 to 60 different cytidines to uridines in chloroplast RNAs, while there are approximately 300 to 600 RNA editing sites in mitochondrial RNAs in land plants [1,8,9,10]. RNA editing has a significant impact on the expression regulation of chloroplast and mitochondrial transcripts in flowering plants, and abnormal RNA editing may lead to disorders in plant growth, development, and reproduction [6,9,11].

Molecular genetic studies during the last three decades have identified a number of nuclear-encoded RNA editing factors that constitute the editosome complex required for organellar RNA editing. Among these, members of the pentatricopeptide repeat (PPR) protein family can directly bind to RNA targets and act as site recognition factors [2,7,12,13,14]. In addition to the large PPR protein family that provides site-specific recognition, members of several other plant protein families have also been identified as components of editosomes, including the multiple organellar RNA editing factor (MORF) proteins/RNA editing factor interacting proteins (RIPs) [4,6,8], the organelle RNA recognition motif (ORRM) family proteins [15,16], the organelle zinc-finger 1 (OZ1) protein [17], and protoporphyrinogen oxidase 1 (PPO1) [18]. MORF2/RIP2 from the MORF/RIP protein family plays an essential role in chloroplast RNA editing, as both knockout and overexpression of MORF2 can widely affect the editing of nearly all sites in chloroplasts [6,19]. Studies have shown that MORF2 can form homologous and heterologous dimers with itself or MORF9 and also interact with many known RNA editing factors [4,6,18,19,20]. MORF2 is believed to play the role of the central “hub” in the chloroplast RNA editing complex. For example, many RNA editing factors, such as the DUA1 and WP2 proteins in rice, have been found to participate in or affect chloroplast RNA editing by interacting with MORF2 [21,22].

The chloroplast ATP synthase is a large, multi-subunit protein complex assembly that is located in the thylakoid membrane. It catalyzes the synthesis of ATP from ADP and inorganic phosphate by utilizing the electrochemical proton gradient established during the photosynthetic electron transport chain [23]. The chloroplast ATP synthase is composed of the catalytic F_1_ head and the F_0_ motor in the membrane. F_1_ consists of three asymmetric αβ heterodimers, which define the catalytic sites, and the central stalk of subunits γ and ε, which are attached to the c-ring. The F_0_ motor consists of the c-ring rotor, subunit a, and the peripheral stalk [24]. ATPC1, the γ subunit of ATP synthase in *Arabidopsis* chloroplasts, is believed to be important in regulating ATPase activity and the flow of protons through the F_0_ complex [23,24]. Deficiency of the γ subunit caused by the *atpC1* mutation leads to a loss of ATP synthesis and unusual non-photochemical chlorophyll fluorescence quenching [25,26].

In this study, we report that disruption of *ATPC1*, which encodes the γ subunit of *Arabidopsis* chloroplast ATP synthase, arrests chloroplast development, resulting in a pale-green phenotype and early seedling lethality. Our results demonstrate that ATPC1 is required for regulating the RNA editing level at multiple sites in chloroplasts. We further show that ATPC1 is involved in RNA editing by interacting with known multiple-site chloroplast RNA editing factors. Based on these results, we propose that ATP synthase γ subunit ATPC1 has a novel role in regulating the editing level of chloroplast transcripts in *Arabidopsis*.

## 2. Results

### 2.1. Phenotypic and Genetic Characterization of the atpc1 Mutant

**Figure 1:**
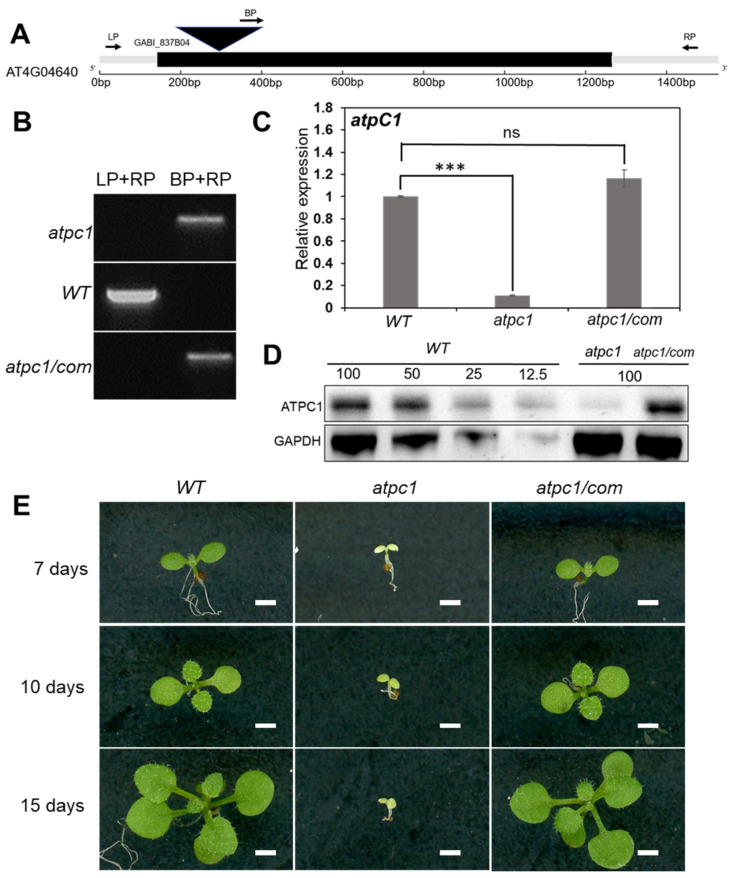
Phenotypic and genetic characterization of *atpc1* mutants. (**A**) The gene structure of *atpC1* and T-DNA insertion position. The black box represents the CDS, the grey boxes represent UTRs, and the black triangle represents the inserted T-DNA. LP, RP, and BP represent primers used in the genotyping. (**B**) Genotyping of the wild-type, *atpc1* mutant, and complementary plants. (**C**) Expression levels of *atpC1* in WT, *atpc1* mutant, and *atpc1/com* plants. Relative expression was calculated using *PP2AA3* (*AT1G13320*) as a reference gene. Data are mean ± SEM from three biological replicates, and asterisks indicate a statistical difference (*** *p* < 0.001) compared with *Col-0* wild type using a two-tailed Student’s *t* test. ns, not significant. (**D**) Immunoblot analysis of ATPC1 protein levels. The lanes were loaded with a series of dilutions as indicated. Immunoblotting against the GAPDH antibody served as the loading control. (**E**) The phenotype of wild-type, *atpc1* and complementary plants. The seedlings were grown on ½ LS + 0.8% agar medium under long-day conditions (16 h light/8 h dark 22 °C) for 7 days, 10 days, and 15 days. The scale bar is 2 mm.

*atpc1* (GABI_837B04) is a mutant derived from the wild-type *Arabidopsis thaliana Col-0* background by a T-DNA insertion in the coding region of the *AT4G04640* gene, resulting in the loss of function of *atpC1*. *atpC1* is an intronless gene, and sequencing results confirmed that the T-DNA insertion was located at 281 bp downstream of the start codon in the *atpc1* mutant (Figure 1A). Homozygous *atpc1* mutant plants were isolated from the progeny of heterozygotes and confirmed by genotyping PCR (Figure 1B). The RNA expression level of *atpC1* in the *atpc1* homozygous mutant dropped to about 10% of the wild type (*Arabidopsis thaliana Columbia-0* ecotype: *Col-0*) (Figure 1C). The protein level of ATPC1 also dramatically decreased in the *atpc1* mutant (Figure 1D). Compared with the wild type, the homozygous *atpc1* mutant plants developed pale-green cotyledons but no primary leaves when grown under standard light conditions and died shortly afterwards (Figure 1E).

As the *atpc1* homozygous mutant died at the seedling stage, this made it impossible to obtain homozygous *atpc1* mutant seeds. To confirm that these phenotypes are caused by the loss of function of the *atpC1* gene, we carried out molecular complementation. A fragment containing the native *atpC1* promoter and the full-length *aptC1* gene fused with a 3×FLAG epitope tag at the 3′ end was cloned and transferred to the pEarleyGate101 binary vector to construct the complementary vector. The vector was introduced into the *atpc1* heterozygous mutant plants via *Agrobacterium tumefaciens*-mediated transformation to obtain complementary transgenic plants (*aptc1/com*) of *atpc1* mutants. We successfully isolated homozygous transgenic plants with the homozygous *atpc1* mutant background in T3 generation plants (Figure 1B). The expression of *atpC1* in the complementary *atpc1/com* plants returned to a similar level as in the wild type (Figure 1C). The protein level of ATPC1 also returned to a similar level to the wild type in the complementary plants (Figure 1D). The complementary *atpc1/com* plants displayed a normal green phenotype compared with the wild type and were able to grow into mature plants (Figure 1E). These results confirm that the phenotype of *aptc1* mutant plants is caused by the loss of function of the *atpC1* gene.

### 2.2. Disruption of atpC1 Results in Abnormal Chloroplast Development in Arabidopsis

**Figure 2:**
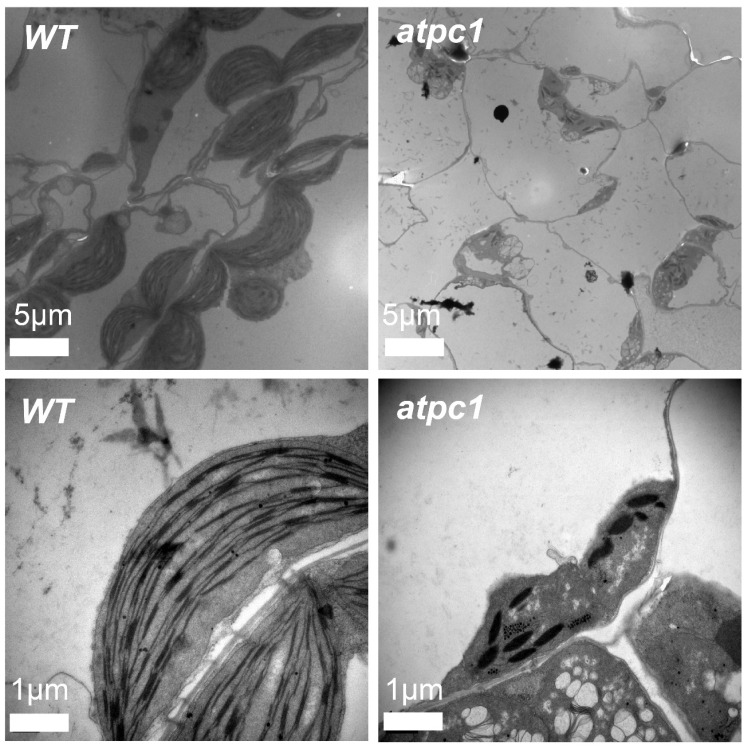
Ultrastructure of chloroplasts in wild-type and *atpc1* mutant seedlings. The leaves of 10-day-old seedlings were used for transmission electron microscopic observation.

The pale-green young seedling and lethal symptoms of the *atpc1* homozygous mutant indicate impaired chloroplast biogenesis and function of the mutant. We observed the ultrastructure of chloroplasts using transmission electron microscopy. The leaf plastids of the *atpc1* mutant are dramatically reduced in number and are irregularly shaped and smaller than wild-type chloroplasts. The thylakoid stacking structure in the chloroplasts of the *atpc1* mutant was disordered, not strictly parallel to each other, and more wrinkled than that of wild-type plants, indicating that the chloroplast development of the *atpc1* mutant was seriously affected. In addition, the chloroplasts of the *atpc1* homozygous mutant lacked starch granule accumulation compared with wild-type plant chloroplasts, indicating that the photosynthesis of the *atpc1* mutant was also greatly impaired. (Figure 2). These results suggest that ATPC1 is essential for the development of pro-plastids into functional chloroplasts.

### 2.3. The ATPC1 Protein Is Localized in Chloroplasts

**Figure 3:**
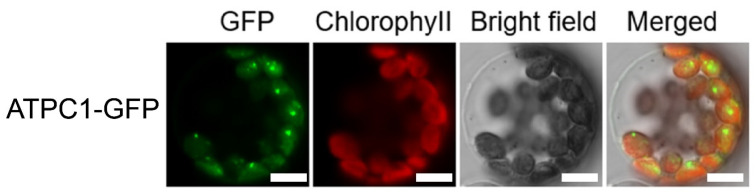
Localization of ATPC1 proteins in *Arabidopsis* protoplasts. The red autofluorescence of chlorophyll was used as an indicator of the localization of chloroplasts. Bright-field images correspond to the protoplast cells. Merged images show the colocalization of GFP with chloroplasts. (Scale bar: 5 μm).

The gene encoding the *Arabidopsis* chloroplast ATP synthase subunit is typically localized in chloroplasts [27]. *Arabidopsis* RNA editing factors also mostly target chloroplasts or mitochondria [1]. To study the subcellular localization of ATPC1 in *Arabidopsis thaliana* cells, we constructed an ATPC1-GFP fusion vector and transiently expressed it in *Arabidopsis* mesophyll protoplasts. Analysis by confocal imaging of *Arabidopsis* protoplasts indicates that the ATPC1-GFP fusion proteins (green fluorescent protein fused to the C terminal of ATPC1) show GFP fluorescence exclusively in chloroplasts, as the GFP co-localizes with the red chlorophyll autofluorescence showing the localization of chloroplasts (Figure 3).

### 2.4. The Loss of Function of atpC1 Significantly Affects the RNA Editing of Multiple Sites in Chloroplasts

**Figure 4:**
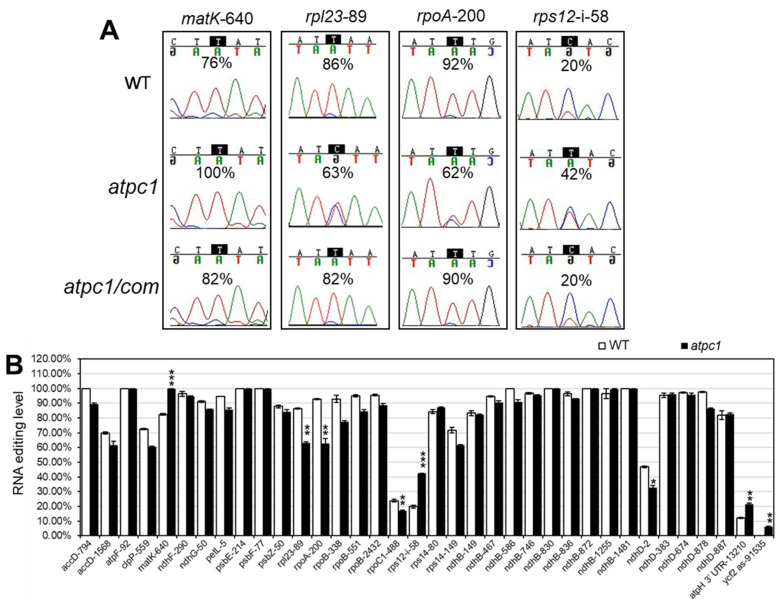
Chloroplast RNA editing profile in the *atpc1* mutant. (**A**) Sequencing chromatogram analysis shows that the RNA editing level in chloroplasts is affected in the *atpc1* mutant (The four sites having ≥20% editing level changes are listed). The peak for C is in blue, the peak for T is in red, the peak for A is in green, and the peak for G is in black. The edited sites are highlighted by dark blocks, and the calculated C to T (equal C to U in RNA) editing levels are labeled. (**B**) The comparison of editing levels of 36 RNA editing sites in *Arabidopsis* chloroplasts between wild-type and *atpc1* mutant plants. The *x* axis indicates the different RNA editing sites. The *y* axis represents the editing level of each site. Data are mean ± SEM from three biological replicates. Asterisks represent the significance level (* *p* < 0.05, ** *p* < 0.01, *** *p* < 0.001) compared with the wild type using a two-tailed Student’s *t* test.

To investigate the effect of the *atpC1* mutation on chloroplast RNA editing, we analyzed the transcripts of 20 chloroplast-encoded genes harboring 36 chloroplast RNA editing sites [28] between WT and *atpC1* by bulk Sanger sequencing of RT–PCR products and RNA sequencing through the Chloroseq pipeline [29,30]. Sanger sequencing results indicated that the *matK*-640, *rpl23*-89, *rpoA*-200, and *rps12*-i-58 sites have ≥20% editing level changes with a *p* value less than 0.05 (from three biological replicates) in the *atpc1* mutant compared to the wild type (Figure 4A,B). The editing level of *matK*-640 and *rps12*-i-58 sites increased from 76% to 100% and from 20% to 42% in the *atpc1* mutant compared to the wild type, respectively. *atpH*-3′UTR-13210 and *ycf2*-as-91535 sites also showed increased editing levels with a *p* value less than 0.05, with *atpH*-3′UTR-13210 increasing from 12% to 22% and *ycf2*-as-91535 increasing from 0 to 6% (Figure 4B). In addition, the editing level of *rpl23*-89, *rpoA*-200, *rpoC1*-488, and *ndhD*-2 sites in the *atpc1* mutant was significantly lower than in the wild type, and the editing level of *rpl23*-89, *rpoA*-200, *rpoC1*-488, and *ndhD*-2 in the *atpc1* mutant reduced by about 23%, 30%, 7%, and 15%, respectively (Figure 4A,B). The Chloroseq analysis revealed similar RNA editing level changes as the Sanger sequencing analysis (Table S1). The remaining RNA editing sites in chloroplasts were not significantly regulated in the *atpc1* mutant (Figure 4B). The editing levels of the four editing sites with ≥20% changes restored the wild-type level in the complementary *atpc1/com* plants (Figure 4A). These results indicate that ATPC1 has a role in regulating the editing levels at specific sites in *Arabidopsis* chloroplasts.

### 2.5. ATPC1 Interacts with Essential Chloroplast RNA Editosome Component MORF2

**Figure 5:**
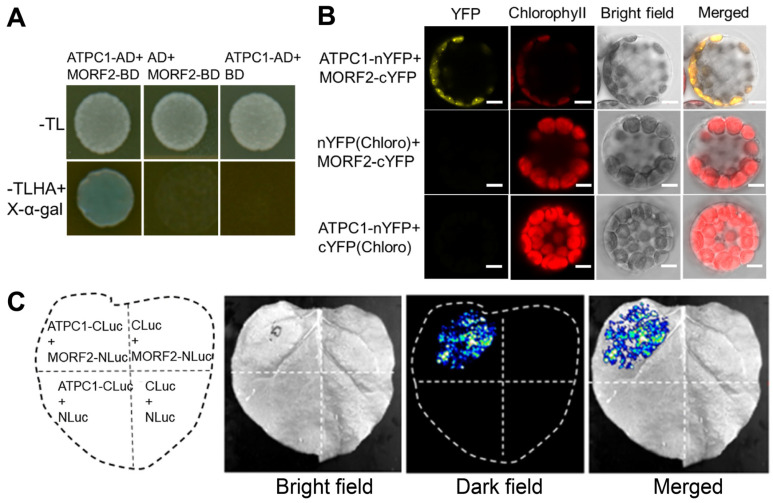
ATPC1 interacts with MORF2. (**A**) Y2H assays show the interaction between ATPC1 and MORF2 in yeast. AD and BD represent the GAL4 activation and DNA binding domains, respectively. -TL and -TLHA/X-α-gal represent the SD/-Trp/-Leu and SD/-Ade/-His/-Trp/-Leu/X-α-gal dropout plates, respectively. The growths of colonies on -TL plates indicate successful co-transformation. The growth as well as the blue color of the colonies on -TLHA/X-α-gal plates showing the reporter gene activity indicate the interaction. (**B**) ATPC1 interacts with MORF2 as shown by the BiFC assay. The co-transformation of ATPC1-nYFP with MORF2-cYFP reconstitutes the YFP signal. Chlorophyll red autofluorescence indicates the localization of chloroplasts. Bright-field images correspond to the protoplast cells. Merged images show the co-localization of YFP and chloroplasts. ATPC1-nYFP/cYFP(Chloro) and MORF2-cYFP/nYFP(Chloro) co-transformations are negative controls. (Scale bar, 5 μm). (**C**) The LCI assay shows the interaction between ATPC1 and MORF2. The co-transformation of APTC1-CLuc and MORF2-NLuc complements the luciferase activity. Target proteins co-transformed with empty plasmids were used as negative controls. The constructs were co-transformed into 4-week-old tobacco leaves by infiltration, and luminescence was monitored after 2 to 3 days of infiltration.

To determine whether ATPC1 plays a direct or indirect role in RNA editing, we performed a yeast two-hybrid assay and found that ATPC1 interacted strongly with MORF2 (Figure 5A). MORF2 is an important component of chloroplast RNA editosomes, and it is required for the editing of almost all sites in plastid RNA [6]. Interaction between ATPC1 and MORF2 was further validated in plant cells by the bimolecular fluorescence complementation (BiFC) assay (Figure 5B) and firefly luciferase complementation imaging (LCI) assay (Figure 5C). For BiFC, coexpression of ATPC1 fused to the N-terminal fragment of YFP (nYFP) and MORF2 fused to the C-terminal fragment of YFP (cYFP), reconstituted YFP fluorescence in *Arabidopsis* mesophyll chloroplasts, and the YFP fluorescence exclusively colocalized with the red autofluorescence of chlorophyll (Figure 5B). As negative controls, ATPC1-nYFP or MORF2-cYFP were co-expressed with chloroplast-localized cYFP or nYFP (targeted by the transit peptide of chloroplast-localized OTP81) [19], respectively, and no YFP fluorescence was observed (Figure 5B). For LCI, coexpression of ATPC1-CLuc (C-terminal luciferase fragment fusion) with MORF2-NLuc (N-terminal luciferase fragment fusion) in tobacco epidermal cells led to a high level of luciferase activity, whereas negative controls showed low or no luciferase activity (Figure 5C). These results confirm the interaction between ATPC1 and MORF2, and also indicate that this interaction occurs in chloroplasts. This result suggests that ATPC1 may participate in chloroplast RNA editing by interacting with other RNA editing factors.

### 2.6. ATPC1 Also Interacts with Other Known Multiple-Site Chloroplast RNA Editing Factors

**Figure 6:**
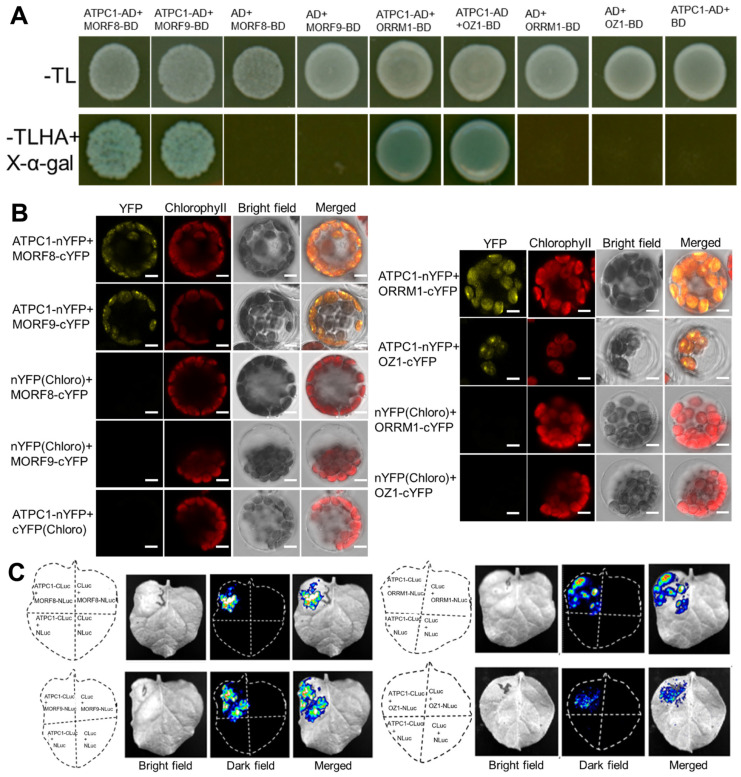
ATPC1 interacts with MORF8, MORF9, ORRM1, and OZ1. The detailed legends are the same as those noted in Figure 5. (**A**) Y2H assays show the interaction of ATPC1 with MORF8, MORF9, ORRM1, and OZ1 in yeast. (**B**) ATPC1 interacts with MORF8, MORF9, ORRM1, and OZ1 in *Arabidopsis* mesophyll chloroplasts, as shown by BiFC assays (Scale bar, 5 μm). (**C**) LCI assays indicate the interaction of ATPC1 with MORF8, MORF9, ORRM1, and OZ1 in tobacco epidermal cells.

As ATPC1 showed a strong interaction with MORF2, we further assayed the possible interaction of ATPC1 with other known chloroplast RNA editing factors. Yeast two-hybrid assays showed that ATPC1 could interact not only with MORF8 and MORF9 but also with ORRM1 and OZ1 (Figure 6A). These interactions in plant cells were also detected by BiFC (Figure 6B) and LCI assays (Figure 6C). For BiFC assays, coexpression of ATPC1-nYFP and MORF8/MORF9/ORRM1/OZ1-cYFP) reconstituted YFP fluorescence in chloroplasts, while no YFP signals were detected in negative controls (Figure 5B). For LCI assays, coexpressions of ATPC1-CLuc with MORF8-NLuc, MORF9-NLuc, ORRM1-NLuc, and OZ1-NLuc all led to high levels of luciferase activity, whereas negative controls showed low or no luciferase activity (Figure 6C). All these results suggest that ATPC1, together with other chloroplast RNA editing factors, may form a large protein complex participating in chloroplast RNA editing.

### 2.7. Differential Expression of Photosynthesis Genes and Chloroplast Genes in the atpc1 Mutant

**Figure 7:**
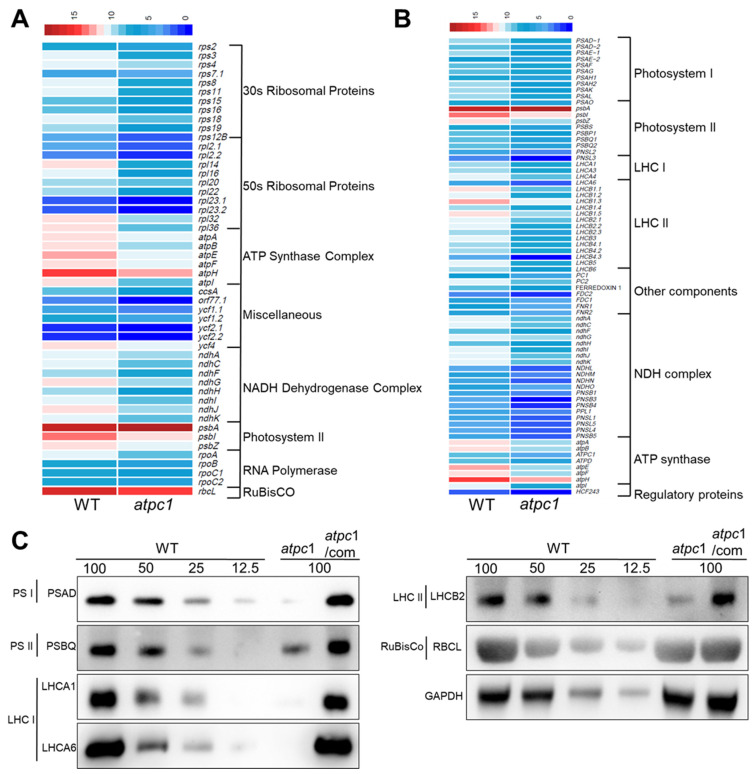
Expression levels of differentially expressed protein-coding chloroplast genes and photosynthetic genes in the *atpc1* mutant. (**A**) The heat map of expression levels of differentially expressed protein-coding chloroplast genes in the *atpc1* mutant and the wild type. The heatmap shows the log2-transformed average RPKM (reads per kilobase of transcript per million mapped reads) of each gene. (**B**) The heat map of expression levels of differentially expressed photosynthetic genes in the *atpc1* mutant and the wild type. The heatmap shows the log2-transformed average RPKM of each gene. The genes with all capital letters are the nucleus-encoded genes while the rest are chloroplast-encoded genes. (**C**) Immunoblot analysis of PSAD, PSBQ, LHCA1, LHCA6, LHCB2, and RBCL. The lanes were loaded with a series of dilutions as indicated. Immunoblotting against the GAPDH antibody served as the loading control.

We also performed differential expression gene analysis in 10-day-old *atpc1* mutants by ribosomal RNA-depleted RNA sequencing. The RPKM (reads per kilobase of transcript per million mapped reads) of each gene is listed in Table S2. We identified 7558 differential expression genes (had a log2-converted fold change ≥ 1 or ≤−1 with a False Discovery Rate ≤ 0.05) in the *atpc1* mutant compared with the wild type (Table S3). Among these, expressions of 4194 genes were repressed, while the remaining 3364 genes had up-regulated expression in the *atpc1* mutant (Table S3). Moreover, the results showed that more than half of the protein-coding chloroplast genes were differentially expressed in the *atpc1* mutant (Figure 7A and Table S4). The expression of plastid-encoded RNA polymerase- (PEP)-dependent chloroplast genes *psbI* and *psbZ* declined in the *atpc1* mutant, while *psbA* had increased expression (Figure 7A). However, the other PEP-dependent chloroplast genes did not differentially express. Meanwhile, many nucleus-encoded RNA polymerase (NEP)-dependent chloroplast genes, including *rpoA*, *rpoB*, *rpoC1*, *rpoC2*, *rps15*, and *ycf1.1* were repressed in the *atpc1* mutants (Figure 7A).

To investigate how the loss of function of *atpC1* affects the photosynthesis of plants, we also analyzed the differential expression of both chloroplast-encoded and nucleus-encoded photosynthesis-related genes in 10-day-old *atpc1* mutants using the RNA-sequencing data. The results showed that more than 60% of photosynthesis-related genes are repressed in the *atpc1* mutant (Figure 7B and Table S5). The majority of nucleus-encoded photosystem I (PSI) genes (e.g., *PSAD*-1, *PSAD*-2, *PSAH*, and *PSAO*), PSII genes (e.g., *PSBP1*, *PSBP2*, and *PSBS*), and light-harvesting complex genes (e.g., *PSI LIGHT HARVESTING COMPLEX GENE 1* (*LHCA1*), *LHCA3*, and *LHCA4*; *PSII LIGHT HARVESTING COMPLEX GENE 1.1* (*LHCB1.1*), *LHCB1.2*, *LHCB3* and *LHCB4.1*) are also repressed in the *atpc1* mutant. This result is consistent with our previous speculation that photosynthesis is inhibited in the *atpc1* mutant. Interestingly, most of the genes encoding ATP synthase subunits (e.g., *atpA*, *atpB*, atpE, *atpF*, *atpH*, *atpI*, *ATPC1*, and *ATPD*) are repressed in the *atpc1* mutant (Figure 7B). We next examined the protein levels of representative thylakoid complex contents, including photosystem I (PSI) (PSAD), PSII (PSBQ), light harvesting complex of PSI (LHCA1 and LHCA6), light harvesting complex of PSII (LHCB2), and Rubisco (RBCL). The content of these representative subunits of these complexes drastically decreased in the *atpc1* mutant (Figure 7C), which was consistent with the results obtained in the gene differential expression analysis.

**Figure 8:**
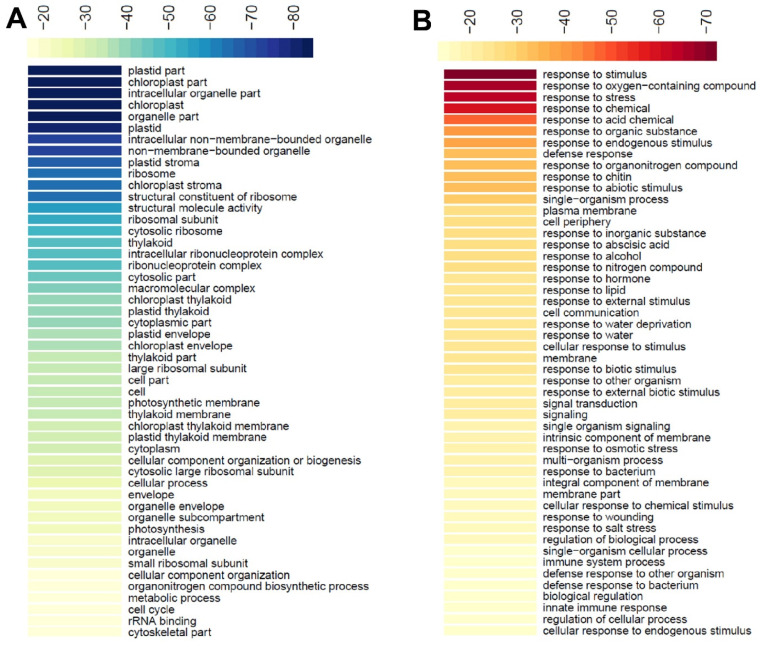
GO term enrichment of differentially expressed genes in the *aptc1* mutant. The color in each cell represents the significance of enrichment based on the Log10-transformed (FDR) value. (**A**) Heat map of top 50 significant enriched GO terms for down-regulated DEGs. (**B**) Heat map of top 50 significant enriched GO terms for up-regulated DEGs.

To find out which biological functions of these differentially expressed genes are mainly involved, we performed GO enrichment analysis on 3364 up-regulated genes and 4194 down-regulated genes, respectively. The results showed that the top 50 enriched GO terms of down-regulated genes were mostly related to the biological function of chloroplasts (Figure 8A) while the top 50 enriched GO terms of up-regulated genes were mainly related to stress responses (Figure 8B). These observations are consistent with the fact that chloroplast development is severely impaired in the *atpc1* mutant.

## 3. Discussion

*atpC1* was initially found to encode the gamma subunit of ATP synthase in *Arabidopsis* chloroplasts [26], but its role in RNA editing of *Arabidopsis* chloroplasts was first reported in this study. Photosynthesis is the process by which plants, algae, and some bacteria convert light energy into chemical energy in the form of glucose and other organic compounds [31]. This process involves a series of biochemical reactions that occur in specialized structures called chloroplasts, which contain the pigment chlorophyll [32]. In this study, we found that the functionally deficient homozygous *atpc1* mutant showed pale cotyledons and inhibited seedling growth, leading to seedling death (Figure 1D). The results of transmission electron microscopy showed that the biogenesis and development of the *atpc1* chloroplast were severely disrupted, and starch granule accumulation was lacking in the *atpc1* chloroplast (Figure 2). This result also often means that the photosynthesis of *atpc1* is greatly impaired. Indeed, the expression levels of photosynthesis genes in the *atpc1* mutant are significantly affected. The functional deficiency of *atpC1* affects the synthesis of ATP synthase in *Arabidopsis* chloroplasts, which in turn leads to plants being unable to obtain sufficient energy for growth, but the role of changes in RNA editing levels in this process remains to be elucidated. We speculate that the synthesis of *Arabidopsis* chloroplast ATP synthetase is affected, which directly affects the energy metabolism process of *Arabidopsis* seedlings. The insufficient energy supply of the plant will lead to the chloroplasts of the plant not being able to carry out normal synthesis and development, which will further impact the photosynthesis of the plant, and eventually lead to the death of the plant.

ATPC1 is targeted to chloroplasts, and its absence results in a complex phenotype, including alterations to transcripts for many chloroplast genes in expression levels as well as RNA editing levels. Among the 36 RNA editing sites in *atpc1* mutant chloroplasts, the editing levels of multiple editing sites show significant changes, including *rpl23*-89, *rpoA*-200, *rpoC1*-488, and *ndhD*-2, which are down-regulated. On the contrary, the editing levels of *matK*-640, *rps12*-i-58, and *atpH*-3′UTR-13210 editing sites are significantly up-regulated, and the editing level of the *ycf2*-as-91535 site increases from 0 to 6% (Figure 4A,B). The editing level changes in the *atpc1* mutant are unlikely to be a secondary effect caused by changes in the transcript itself because editing level changes are only observed at specific sites, and the editing levels of different sites in the same transcript are not all affected in the same pattern by the *atpc1* mutation. For example, *ndhD*-2 sites have significantly decreased editing in the *atpc1* mutant, but other editing sites in the *ndhD* genes are not affected (Figure 4B). Moreover, our RNA sequencing data showed that genes for *atpc1*-mutation-affected sites associated site-recognition PPR proteins and multiple-site plastid RNA-editing factors were not differentially expressed (Table S3). Thus, changes in the RNA-editing level in the *atpc1* mutant do not seem to be caused by secondary effects of changes in gene expression. Combined with our differential expression gene analysis results, we found that *rpl23, rpoA*, *rpoC1, atpH*, and *ycf2.1*, which contain the *rpl23*-89, *rpoA*-200, *rpoC1*-488, *atpH*-3′UTR-13210, and *ycf2*-as-91535 editing sites, respectively, showed decreased expression in the *atpc1* mutant (Table S4). Moreover, we found that the expressions of most ATP synthase subunit-coding genes (e.g., *atpA*, *atpB*, atpE, *atpF, atpH, atpI, ATPC1,* and *ATPD*) were repressed in the *atpc1* mutant (Figure 7B). These results suggest that the loss of function of *ATPC1* with abnormal energy metabolism causes effects on RNA editing and the development of *Arabidopsis* chloroplasts. Previous studies also showed that abnormal energy production in chloroplasts and mitochondria caused by overexpression of *AtPAP2* led to altered levels of RNA editing at some sites [10].

In this study, we verified the interaction between ATPC1 and multiple-site plastid RNA-editing factors, including MORF2, MORF8, MORF9, ORRM1, and OZ1, using yeast two-hybrid, BiFC, and LCI assays (Figure 5 and Figure 6). However, *atpC1* mutation only regulates the editing of specific sites with distinctive changing patterns. Multiple-site plastid RNA-editing factors in chloroplasts such as MORFs, ORRM1, and OZ1 usually share common editing sites and selectively interact with each other; this may reflect the need for the interaction of several editing factors to achieve editing as well as a competition binding between different RNA editing factors. MORF proteins can form homomers or heteromers and also selectively interact with other RNA editing factors in RNA editosomes [4]. MORF proteins have been reported to bind PLS-class PPR proteins to enhance their affinity to target RNAs [33]. Furthermore, MORF2 and MORF9 function as holdase chaperones to facilitate the folding of their client proteins and enhance their activities in chloroplasts to control various processes during chloroplast development, including RNA editing [34]. Monomers or multimers of other MORFs may be able to partially substitute for MORF2. It is possible that the selective binding of ATPC1 to multiple-site plastid RNA-editing factors affects the multimerization of MORFs, or the chaperon activity, or the interaction of MORFs with other RNA editing factors to selectively regulate RNA editing level. For example, the RNA editing level at the *rps12*-i-58 site increases in the *atpc1* mutant, and this increase is also observed in both the *MORF8/RIP1* activation T-DNA insertion *rip1* mutant and transient silencing plants [8]. As ATPC1 can interact with MORF8/RIP1, it is possible that the interaction between ATPC1 and MORF8/RIP1 influences the activity of MORF8/RIP1 in the editing of *rps12*-i-58. Interactions between ATPC1 and other editing factors may play a regulatory role that finetunes the RNA editing of specific sites in chloroplasts. Further determination of why ATPC1 is recruited to affect the RNA-editing level at specific sites and how this specific recruitment is organized will help to elucidate the finely tuned process of RNA editing in chloroplasts.

## 4. Materials and Methods

### 4.1. Plant Materials and Growth Condition

*Arabidopsis Col-0* wild-type seeds were from our lab seed stock. *atpc1* T-DNA insertion mutant (GABI_837B04) was ordered from ABRC (https://abrc.osu.edu/ (accessed on 20 January 2020)) [35]. Seeds were surface-sterilized using chlorine gas for 4 h and placed on a 1/2 Linsmaier and Skoog (LS) basal salts with buffer medium (LSP03, Caisson Labs, Smithfield, UT, USA) plate with 0.8% micropropagation type-1 agar (A038, Caisson Labs, Smithfield, UT, USA). After a 4-day stratification in the dark at 4 °C, plates were placed in long day conditions under 100 µmol·m^−2^·s^−1^ light intensity at 22 °C for 10 days. For transgenic plant planting, plants were grown in long day conditions (16 h light/8 h dark) under 100 µmol·m^−2^·s^−1^ light intensity at 22 °C. The tobacco seed used in the LCI experiment was from our lab seed stock. The tobacco plants were grown in long day conditions with 100 μmol·m^−2^·s^−1^ light intensity at 23 °C for 3–4 weeks.

### 4.2. Mutant Genotyping

Homozygous T-DNA insertion mutant lines were identified using *atpC1* gene-specific primers (LP: 5′-TCCACTAATACAACGCCACG-3′; RP: 5′-TCCATCTCAATGTCCAACCC-3) and T-DNA left-border primer (BP: 5′-ATAATAACGCTGCGGACATCTACATTTT-3′). PCR was carried out using the 2×M5 Taq HiFi PCR mix (with blue dye) (MF002, Mei5bio, Beijing, China). The following thermal condition was used: 94 °C for 5 min, 35 cycles of 94 °C for 30 s, 52 °C for 45 s, 72 °C for 1 min.

### 4.3. Transmission Electron Microscopy Analysis

Wild-type and *atpc1* mutant tissues were fixed in 2.5% glutaraldehyde at 4 °C for 16 h. The samples were then rinsed and incubated overnight in 1% OsO_4_, followed by staining with uranyl acetate, dehydration in an ethanol series, and embedding in Spurr’s medium prior to ultrathin sectioning. Subsequently, the samples were re-stained with uranyl acetate and observed under a Hitachi H-7650 transmission electron microscope.

### 4.4. Plant Transformation and Expression Level Detection

For the complementary experiment, the whole fragment containing the native promoter of *atpC1*, the gene body of *atpC1* (without stop codon) and the coding sequence of the 3×FLAG tag was amplified by PCR using Q5 Hot Start High-Fidelity 2×Master Mix (M0494S, NEB, Ipswich, MA, USA). The following thermal condition was used: 98 °C for 3 min, 33 cycles of 98 °C for 10 s, 52 °C for 30 s, 72 °C for 1 min. The PCR fragment was then transferred to the modified binary vector pEarleyGate101 [36] digested by restriction enzymes MluI and SpeI (NEB, Ipswich, MA, USA). In the final vector, the *atpC1* gene (without stop codon) was driven by native promoter and fused with a 3×FLAG tags in the C-terminal. Plasmids were then introduced into *Agrobacterium tumefaciens* strain GV3101 to transform the *atpc1* T-DNA insertion heterozygous mutant plants using the floral-dip method [37]. Transgenic plants with Basta resistance selected on 1/2 LS medium plate with 10 μg/mL Basta (Sigma Aldrich, Darmstadt, Germany) were further propagated, and T3 homozygous seeds were chosen for further study. At least two independent lines were examined with similar results, and one representative line was shown. The expression level of the *atpC1* gene was examined by quantitative reverse transcription–PCR (qRT–PCR). Total RNA was isolated from whole seedlings (grown under long day conditions with 100 µmol·m^−2^·s^−1^ light intensity at 22 °C for 10 days as indicated) using the RNAprep Pure Plant Kit (DP432, Tiangen, Beijing, China). The first-strand cDNA was synthesized using the HiScript III 1st Strand cDNA Synthesis Kit (+gDNA wiper) with both Oligo (dT)_20_VN and random hexamer primers (R312-02, Vazyme, Nanjing, China) added. qRT–PCR was performed using a Bio-rad CFX Connect Real-Time PCR Detection System with ChamQ Universal SYBR qPCR Master Mix (Q711-02, Vazyme, Nanjing, China) with three biological replicates. Expression levels for all assayed genes were normalized using *PP2AA3* (*AT1G13320*) [38] as the internal control. The qRT–PCR analysis primers for *atpC1* were *atpC1*-qF: 5′-ATCTCGGTTAGGTTGTTC-3′ and *atpC1*-qR: 5′-ATCATCTGCTGCTTAGTC-3′.

### 4.5. RNA Isolation and RNA Editing Analysis

Total RNA was isolated from whole seedlings (grown under long day conditions with 100 µmol·m^−2^·s^−1^ light intensity at 22 °C for 10 days as indicated) using the RNAprep Pure Plant Kit (DP432, Tiangen, Beijing, China). The first-strand cDNA was synthesized using the HiScript III 1st Strand cDNA Synthesis Kit (+gDNA wiper) with both Oligo (dT)_20_VN and random hexamer primers (R312-02, Vazyme, Nanjing, China) added. PCR fragments containing chloroplast RNA editing sites were obtained with specific primers surrounding editing sites by RT–PCR using the OneTaq^®^ 2×Master Mix with Standard Buffer (M0482L, NEB, Ipswich, MA, USA). The following thermal condition was used for RT–PCR: 94 °C for 3 min, 33 cycles of 94 °C for 30 s, 52 °C for 45 s, 72 °C for 1 min. PCR products were then purified and used as templates for Sanger DNA sequencing (carried out by Sunya, Hangzhou, China). The amplifying and sequencing primer for each site is listed in Table S6. The “C” to “T” (equal C to U in RNA) editing level of each site was measured by the relative height of the peak of the nucleotide in sequence chromatograms and calculated by the height of “T” divided by the sum of the height of “T” and “C”. Statistical significances were calculated using a two-tailed Student’s *t* Test in Excel. For RNA editing level revealed by RNA sequencing data, we applied the Chloroseq analysis pipeline; the detailed RNA sequencing data processing steps were as below. The Chloroseq analysis pipeline was carried out as previously described [29,30] and manually examined using bam files in the IGV browser [39].

### 4.6. Subcellular Localization

For subcellular localization assay, the CDS of *atpC1* without the stop codon was cloned by PCR using the 2×Phanta Max Master Mix (P525-01, Vazyme, Nanjing, China) and transferred to target plasmids: pUC19-EGFP. Transfection-grade plasmid DNA was prepared using the QIAGEN Plasmid Maxi Kit (12163, Qiagen, Hilden, Germany). Protoplasts were extracted from *Arabidopsis Col-0* wild-type plants grown under short day (12 h light/12 h dark) conditions for 21 days. A weight of 20 μg of plasmids were transformed into *Arabidopsis* protoplasts. Protoplasts were then incubated under constant 100 µmol·m^−2^·s^−1^ light at 22 °C for 16–20 h, and the fluorescence signal was determined using a Zeiss LSM 880 confocal laser scanning microscope.

### 4.7. Yeast Two-Hybrid Assay

The yeast two-hybrid assay was performed following the manual of the Matchmaker™ Gold Yeast Two-Hybrid System (Takara Bio, San Jose, CA, USA) with modifications. Generally, the CDS of each gene was cloned by PCR using 2×Phanta Max Master Mix (Vazyme, Nanjing, China) and vectors were linearized by restriction enzymes NdeI and BamHI. Then, insert genes were transferred to target plasmids using the 2×Seamless Cloning Kit (D7010M, Beyotime, Shanghai, China). Combinations of GAL4 DNA binding domain (pGBKT7) and GAL4 activation domain (pGADT7) fusions of corresponding genes were co-transformed into the yeast strain Y2HGold (Takara Bio, San Jose, CA, USA). Co-transformants were placed on SD/–Leu/–Trp dropout plates under 30 °C in the dark for 5 days to verify successful co-transformation, and then on SD/–Ade/–His/–Leu/–Trp/X-α-Gal dropout plates under 30 °C in dark for 5 days to verify the interaction.

### 4.8. Bimolecular Fluorescence Complementation Assay

For BiFC assays, the CDS of each target gene without the stop codon was cloned and transferred to target plasmids: pSPYNE173 for N-terminus YFP fusion or pSPYCE(M) for C-terminus YFP fusion. Vectors were digested by XbaI and SalI. Transfection-grade plasmid DNA was prepared using the QIAGEN Plasmid Maxi Kit (Qiagen, Hilden, Germany). For control plasmids, the coding sequence of the transit peptide of OTP81 was cloned by PCR and transferred to pSPYNE173 or pSPYCE(M) for N- or C-terminal YFP fragment fusions targeting chloroplasts, respectively. A weight of 20 μg of each transfection-grade plasmid was co-transformed into *Arabidopsis* protoplasts isolated from *Col-0* that were grown in short day conditions under 100 µmol·m^−2^·s^−1^ light intensity at 22 °C. Protoplasts were then incubated under constant 100 µmol·m^−2^·s^−1^ light at 22 °C for 16–20 h and the fluorescence signal was determined using a Zeiss LSM 880 confocal laser scanning microscope.

### 4.9. Firefly Luciferase Complementation Imaging (LCI) Assay

For LCI assays, the CDS of each target gene without the stop codon was cloned and transferred to target plasmids: pCAMBIA1300-NLuc for N-terminal luciferase fragment fusion or modified pCAMBIA1300-CLuc (CLuc is fused to the C-terminal of each gene) for C-terminal luciferase fragment fusion [40]. The plasmids were transformed into the GV3101 *Agrobacterium* strain. After collection by centrifugation, the *Agrobacterium* cells were resuspended in injection buffer (10 mM MgCl_2_, 10 mM MES, pH 5.7, 200 μM Acetosyringone) and kept at room temperature for 4 h. Then, *Agrobacterium* cells with plasmid combinations and with P19 were mixed in a 1:1:1 ratio, and 50 μL of the mixture was injected into tobacco leaves. The injected tobacco plants were then incubated for 60 h. Finally, the tobacco leaves were sprayed with a D-luciferin (Biosynth, Staad, Switzerland) solution at a final concentration of 1 mM, and the luminescence intensity was observed after keeping the leaves in the dark for 7 min.

### 4.10. RNA Sequencing and Data Analysis

Total RNA samples for ribosomal RNA-depleted RNA sequencing were prepared as used in the RNA editing analysis. Three biological replicates were used for RNA-seq analysis. Total amounts and integrity of RNA were assessed using the RNA Nano 6000 Assay Kit of the Agilent Bioanalyzer 2100 system. Ribosome RNA depletion and RNA sequencing library construction were carried out by Novogene (Beijing, China). Paired-end sequencing was performed on the Illumina NovaSeq 6000 sequencing platform in Novogene. Fastp was used for the quality control of sequencing data [41]. Hisat2 was used for aligning the raw reads to the *Arabidopsis* reference genome (TAIR10) [42,43]. Gene-level raw counts were generated using featureCounts [44]. Raw counts were applied to the bioconductor package edgeR [45] in R language to obtain the reads per kilobase of transcript per million mapped reads (RPKM) of each gene and to identify differentially expressed genes (DEGs). When identifying DEGs, a gene was retained only if it was expressed at a count-per-million (CPM) above 0.5 in at least two samples. Genes which had a log2-converted fold change ≥1 or ≤−1 with a false discovery rate (FDR) ≤ 0.05 were considered as DEGs. The gene ontology (GO) term enrichment was analyzed using agriGO v2.0 (http://systemsbiology.cau.edu.cn/agriGOv2/ (accessed on 16 February 2023)) [46] and terms with a FDR < 0.01 were retained for further analysis.

### 4.11. Immunoblot Analysis

Total proteins were isolated from whole seedlings (grown under long-day conditions with 100 µmol·m^−2^·s^−1^ light intensity at 22 °C for 10 days as indicated). Proteins were separated by 4–12% SDS-PAGE gel. After the proteins were transferred electrophoretically onto the nitrocellulose filter membrane, the membrane was incubated with corresponding antibodies. Anti-ATPC1 (AS08312, Agrisera, Vännäs, Sweden), anti-GAPDH (K90002P, Solarbio, Beijing, China), anti-PSAD (PHY0056A, PhytoAB, San Jose, CA, USA), anti-PSBQ (PHY2346A, PhytoAB, San Jose, CA, USA), anti-LHCA1 (PHY0043A, PhytoAB, San Jose, CA, USA), anti-LHCA6 (PHY0470S, PhytoAB, San Jose, CA, USA), anti-LHCB2 (AS01003, Agrisera, Vännäs, Sweden), and anti-RuBisCo (AG5359, Beyotime, Shanghai, China) antibodies were used for immunoblot analysis. GAPDH was used as the reference protein to determine the amount of protein loaded.

## Data Availability

All data generated or analyzed during this study are included in the published article and Supplementary Files.

## Supplementary Materials

The supporting information can be downloaded at: https://www.mdpi.com/article/10.3390/ijms24119203/s1.
